# Endosonography: New Developments in 2006

**DOI:** 10.1100/tsw.2007.28

**Published:** 2007-03-02

**Authors:** Marc Giovannini

**Affiliations:** Endoscopic Unit, Paoli-Calmettes Institut 232 Bd St-Marguerite, 13273 Marseilles-Cedex 9, France

**Keywords:** 3D EUS, US contrast agents, elastosonography

## Abstract

Recent progress of the data processing applied to ultrasound (US) examination made it possible to develop new software. The US workstation of the last generation thus incorporated a computer into their center that allowed a very precise treatment of the US image. This made it possible to work out new images like three-dimensional (3-D) US, the US of contrast-harmonic associated with the intravenous injection with product with contrast for US, and finally even more recently, elastography. These techniques, currently quite elaborate in percutaneous US, are to be adapted and evaluated with echoendoscopy (EUS).

We thus will approach the 3-D EUS successively, then the contribution of the products of contrast for US with the pancreatic EUS, and finally, elastography guided by endosonography.

## THREE-DIMENSIONAL ENDOSCOPIC ULTRASOUND

Recent development of the probes of electronic echoendoscopy (EUS) connected to the ultrasound (US) machine of the last-generation HITACHI 6500 or 8500 with three-dimensional (3-D) software built in currently makes it possible to produce EUS images very easily. The acquisition of the images is very fast, approximately 30 sec; the images in 2-D are rebuilt in six planes different to that of the computer from the US machine. They are returned in a 3-D volume of cubic form, thus making it possible to build transverse and longitudinal cuts of the six faces of the cube (Fig. 1).

The fast acquisition of the images makes it possible to eliminate all the artefacts, in particular on the level of the mediastinum (Fig. 2), those induced by cardiac movements and breathing. It is rather easy, by a simple movement of shrinking the endoscope, if one uses radial echoendoscope EG 36 UR or by a rotational movement of 360° if one uses the EG 38 UT, to carry out 3-D rebuilding of EUS images.

### Basic Principles of 3-D US

Two types of systems have been developed, making use of either a series of 2-D images produced by 1-D arrays, or 2-D arrays to produce 3-D images directly. Two criteria must be met to avoid inaccuracies: the

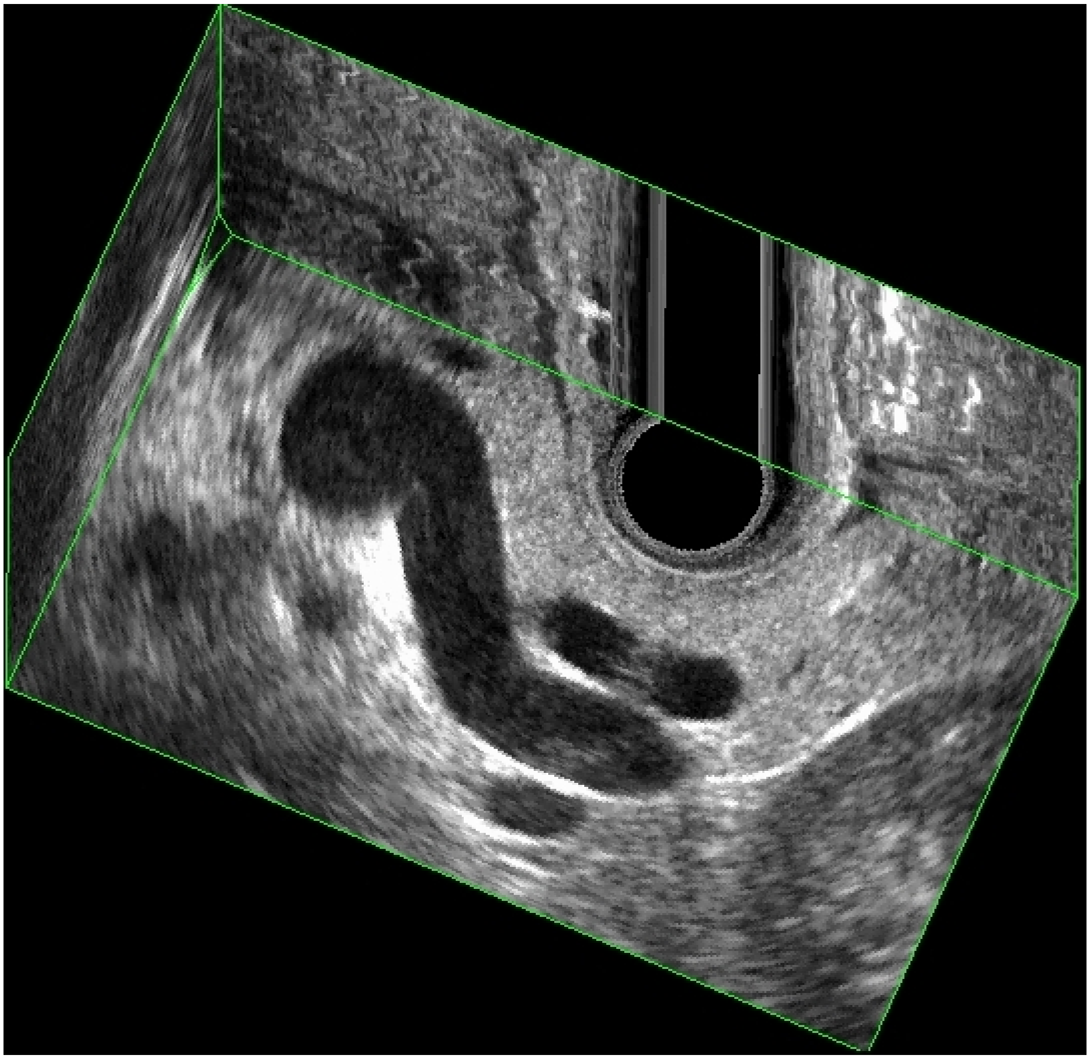

**FIGURE 1**. Normal 3-D reconstruction of normal pancreas using an electronic radial probe (Pentax-EG 3670UKR).

relative position and angulation of the acquired 2-D images must be known accurately; and the images must be acquired rapidly and/or gated to avoid artefacts due to respiratory, cardiac, and involuntary motion.

- Tracked free-hand systems — The operator holds an assembly composed of the transducer and an attachment, and manipulates it over the anatomy. Two-dimensional images are digitized as the transducer is moved while meeting two criteria: the exact relative angulation and position of the US transducer must be known for each digitized image; and the operator must ensure that no significant gaps are left when scanning the anatomy.
- 3-D reconstruction — The 3-D reconstruction process refers to the generation of a 3-D image from a digitized set of 2-D images. The approach used was the voxel-based volume. The 2-D images are built into a 3-D voxel-based volume (3-D grid) by placing each digitized 2-D image into its correct location in the volume. The main advantage was that noninformation was lost during the 3-D reconstruction and a variety of rendering techniques were possible, but large data files are generated.

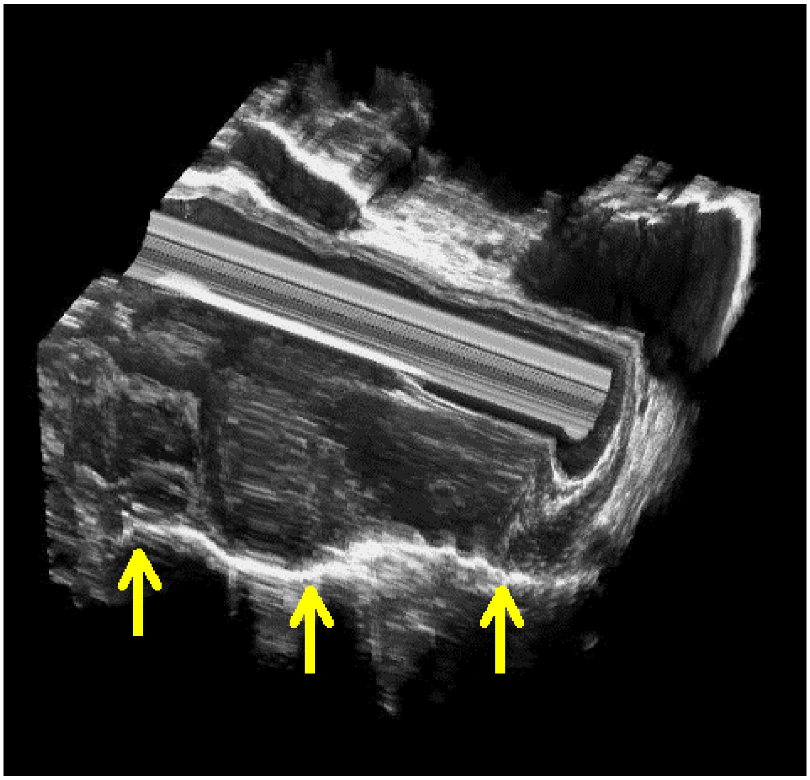

**FIGURE 2.** Three-dimensional reconstruction of an esophageal cancer usT4 (pleura involvement: arrows) using a radial electronic probe (Pentax-EG 36UR).

- Visualization of 3-D US images — The ability to visualize information in the 3-D image depends critically on the rendering technique. Three basic types are being used:
  - Surface-based viewing technique — An operator or algorithm identifies boundaries of structures to create a wire-frame representation. These are shaded and illuminated so that surfaces, structures, or organs are visualized.
  - Multiplane viewing techniques — Orthogonal views: three perpendicular planes are displayed simultaneously and can be moved or rotated; polyhedron: the 3-D image is presented as a multisided volume (polyhedron). The appropriate US image is "painted" on each face of the polyhedron, which can be manipulated.
  - Volume-based rendering techniques — The 3-D image is projected onto a 2-D plane by casting rays through the 3-D image. The voxel values intersected by each ray can be multiplied by factors and summed to produce different effects: multiply by 1 and then add to form a radiographic-like image, multiply by factors to produce translucency; or display only the voxel with the maximum intensity along each ray.

## Clinical Impact of 3-D EUS

The important point is to know the real impact of this new technique in human pathology. Currently, it arises that the contribution of this new type of imagery is only interesting in tumoral pathology of the rectum. Indeed, the first studies show that it is much easier to visualize the mesorectum and its limits in 3-D EUS than in 2-D EUS (Fig. 3). These results have an important impact on therapeutic decision making in

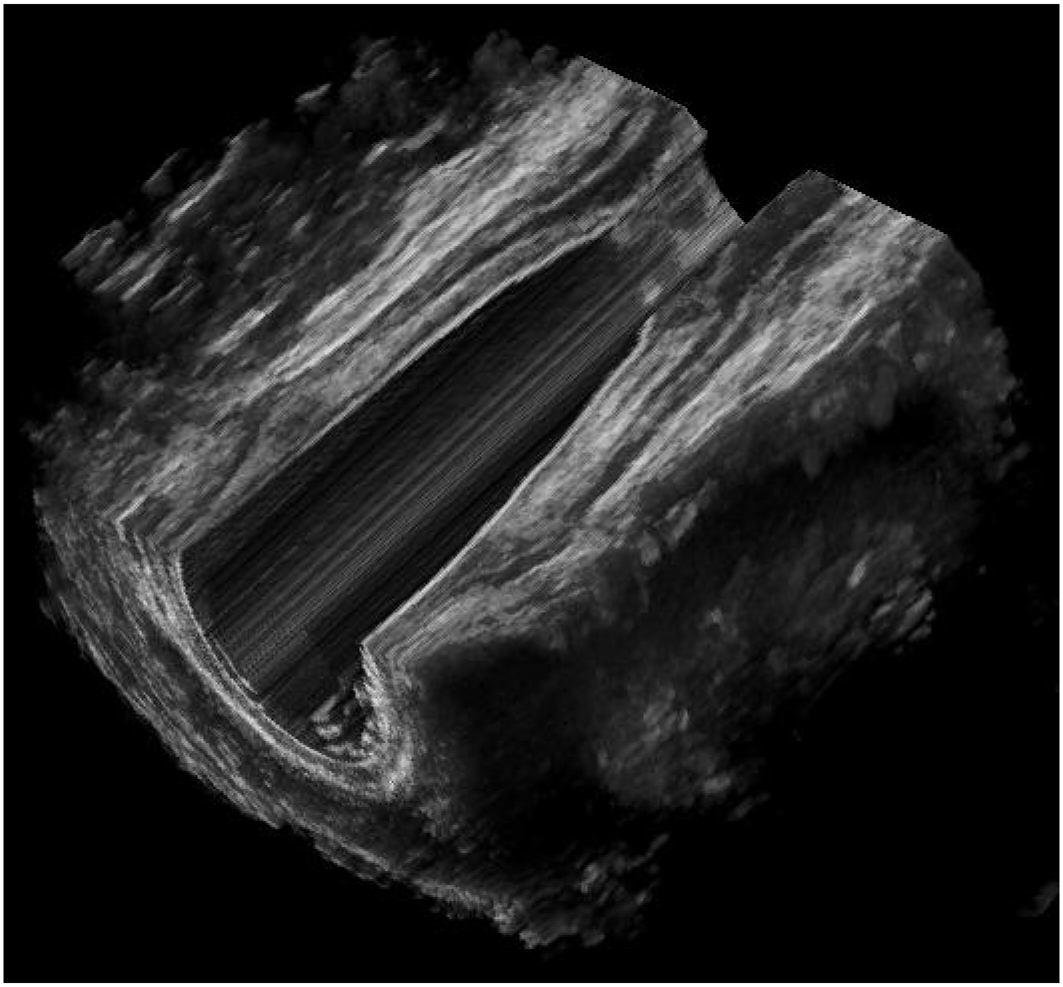

**FIGURE 3**. Three-dimensional reconstruction of normal rectum using a rigid radial probe (Hitachi RW 54).

particular when the tumor involve entirely the mesorectum and the fascia recti (Fig. 4). These rectal cancers have very bad outcomes , approaching metastatic rectal cancer. In addition, the 3-D EUS seems to bring a better reliability with endorectal EUS for the assessment of rectal cancer[1]. This was shown in several studies, in particular, the studies of Odegaard[2]. It would also seem that pancreatic 3-D EUS makes it possible to better appreciate the tumoral infiltration on the level of the porto-spleno-mesenteric confluence of pancreatic cancers (Fig. 5). There are still few data in the literature[3], assessment of venous extension would seem more precise in 3-D pancreatic EUS. In conclusion, to date, it would seem that 3-D EUS brings more information than the conventional EUS to the level of cancers of the rectum. Other studies are necessary to specify the exact place of the 3-D pancreatic EUS in the staging of pancreatic cancer.

## Our Experience in 3-D Endorectal US

We have performed 35 3-D endorectal (ER) US examinations using this new software[3]. The indication of ERUS was a local staging of rectal cancer in 35 cases. Before 3-D ERUS scanning, a standard ERUS was realized. Three-dimensional rectal examination was performed using a radial electronic rigid probe. The 3-D

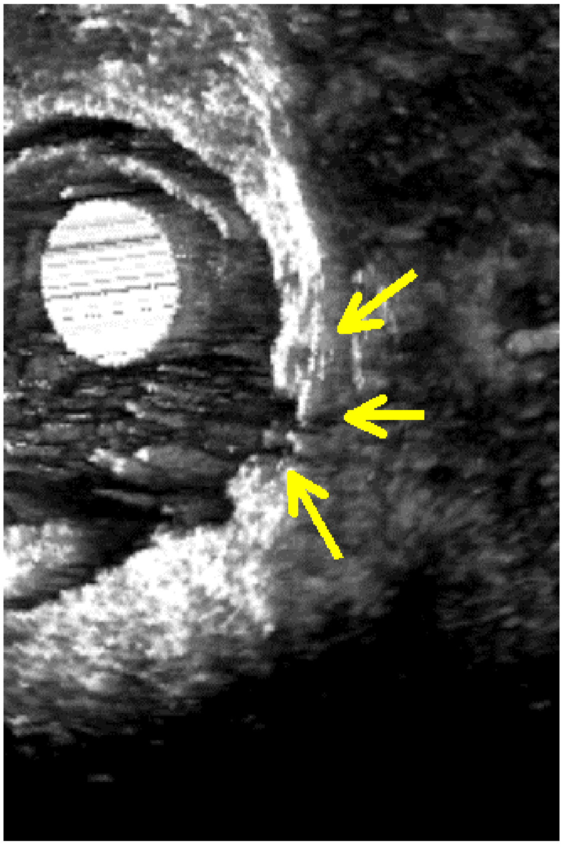

**FIGURE 4.** Rectal cancer with complete involvement of mesorectum (usT4) (arrows).

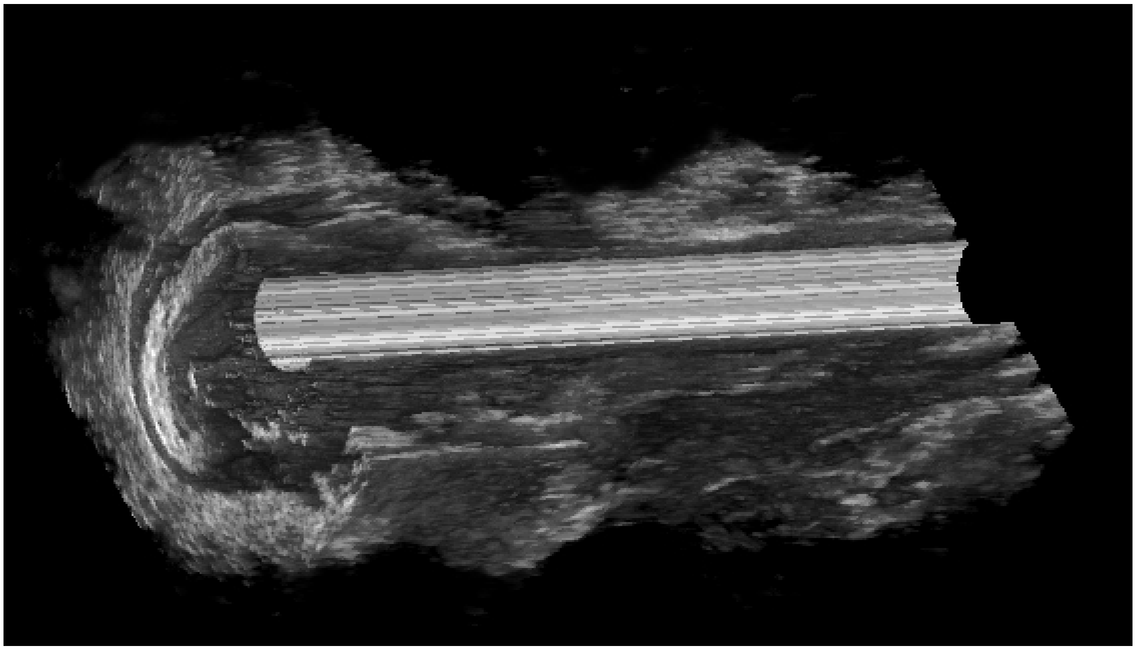

**FIGURE 5.** Pancreatic cancer of the body of the pancreas with portal vein involvement; 3-D reconstruction using a linear probe (Pentax-EG38UT).

software was included in the new US scanning machine (HITACHI 6500 or 8500) and permitted a reconstruction of the 2-D ERUS pictures in six different scans. Acquisition time is very quick, around 10–25 sec. Acquisition time was a function of the number of images recorded.

Using 2-D and 3-D ERUS, 35 rectal cancers were assessed. All tumors were located in the middle and lower part of the rectum and stenotic tumors were excluded from this study. In all cases, 3-D ERUS was possible; 2-D ERUS data classified the tumor in T1N0 (2 cases), T2N0 (3 cases), T3N0 (15 cases), T3N1 (12 cases), and T4N1 (3 cases). It was impossible using 2-D images to determine precisely the degree of involvement of the mesorectum (more or less than 50%). No difference was shown using 3-D ERUS for the superficial tumor (T1 and T2N0), but in 6/15 patients classified T3N0, 3-D ERUS showed malignant lymph nodes, which was confirmed surgically in 5/6 cases (Table 1).

**TABLE 1**
**Correct Findings Using 3-D ERUS**

| Rectal Cancer | T1/T2N0 | T3N0 | T3N1 | T4N1 |
|---|---|---|---|---|
| 2-D EUS | 5 | 15 | 12 | 3 |
| 3-D EUS | 5 | 9 | 18 | 3 |
| Surgical findings | 4 | 6 | 22 | 3 |

On the other hand, 3-D EUS allowed us to determine precisely the degree of infiltration of the mesorectum (Fig. 4) in all cases and showed a quite complete invasion of it in eight cases. These findings were confirmed in all cases by the surgical data. To summarize for rectal cancer, 2-D ERUS correctly assessed 25/35 rectal tumors (71.4%) for T and N classification, and 3-D EUS increased these results to 31/35 correct evaluation (88.6%).

If you compare the 2-D and 3-D accuracy in T staging (T1-2 vs. T3-T4), there was no difference (34/35 accurate staging using the two techniques), but patients classified 3-D T3N0 developed less liver metastasis than those classified 2-D T3N0 (1/9 vs. 6/15, 11 vs. 40%, $p = 0.002$). This difference was due to the fact that 7/15 of 2-D T3N0 rectal cancers are pT3N1 on the resected specimen. On the other hand, of patients in whom 3-D ERUS showed a large mesorectum infiltration (more than two-thirds of the thickness of the mesorectum), 8/35 developed more metastasis (liver or peritoneal carcinomatosis)(4/8 vs. 7/27, 50 vs. 26%, $p < 0.001$).

## PANCREATIC EUS AND US CONSTRAST AGENTS

Diagnosis between adenocarcinomas and nodular chronic pancreatitis is problematic. All methods of diagnosis are limited. Histology is the standard, but even biopsy can be difficult because cancers can produce a marked fibrotic reaction or necrosis, and give false results. For endoscopic retrograde cholangiopancreatography (ERCP), sensitivity and specificity are, respectively, 85 and 66% when there is a stenosis of the main pancreatic duct[4]. Magnetic resonance cholangiopancreatography (MRCP) has a similar sensitivity and specificity for detecting pancreatic cancer or chronic pancreatitis as that of ERCP.

Nevertheless, sensitivity is yet perfectible and MRCP gives a correct differentiation between malignant and benign lesions in 58% of cases[5,6]. MRCP remains an expensive procedure, is time consuming, and is available only in a few centers.

There are few studies about contrast-enhanced EUS (CE-EUS). In one of the first studies, Bhutani et al.[7] evaluated the utility of SHU508 A (Levovist®) and concluded it could potentially improve the accuracy of EUS in the diagnosis of malignant vascular invasion, in detection of occult pancreatic neoplasms, and in the diagnosis of vascular thrombosis. Subsequently, Hirooka et al.[8] studied the presence or absence of enhancement of different lesions with Albunex® in 37 patients. An enhancement of the lesion was observed in 100% of the patients with islet cell tumor (Figs. 6A and B), in 80% with intraductal papillary mucinous tumor (IPMT), in 75% with chronic pancreatitis (Figs. 7A and B), and no enhancement effect was observed in the patients with carcinoma (Fig 8). All patients underwent angiography, and comparison between images of CE-EUS and angiographic images showed similar results, except for three patients (two IPMT and one chronic pancreatitis) in whom angiograms were hypovascular, but enhancement effect was observed on EUS images. Finally, Becker et al.[9] showed their experience in 23 patients with another contrast agent (FS 069 Optison®) and evaluated CE-EUS as a method of differentiating inflammation and carcinoma based on perfusion characteristics. Markedly hyperperfused lesions were considered as inflammatory pseudotumors, whereas hypoperfused lesions compared to surrounding tissue were considered as carcinomas. Sensitivity for differentiation of pancreatic carcinoma vs. inflammatory changes was 94%, specificity 100%, positive predictive value (PPV) 100%, negative predictive value (NPV) 88%. These results are very similar to ours[3] (sensitivity 90.9%, specificity 88.8%, PPV 88.2%, and NPV 91.4%). In our study[3], we also studied hyperechoic lesions (supposed not to be a pancreatic adenocarcinoma), and sensitivity was 88.8%, specificity 90.9%, PPV 91.4%, and NPV 88.2%. In future, CE-EUS could allow us to have a direct and reliable result (malign or not) without waiting several days for histological findings. Perhaps it could also save time and money in limiting the use of expensive EUS needles.

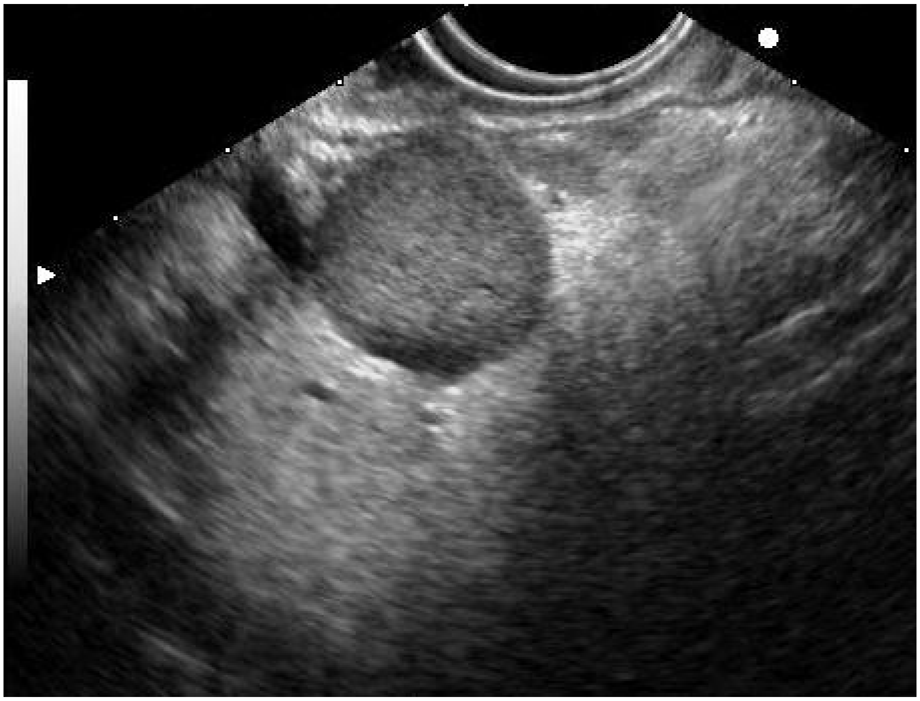

A

**FIGURE 6**. (A) Endocrine tumor of the head of pancreas; (B) enhancement of vascularization after sonovue injection.

CE-EUS could be an interesting complement to EUS fine needle aspiration (FNA) concerning diagnosis accuracy. EUS FNA sensitivity and diagnosis accuracy are, respectively, 75–92% and 79–92%[9,10,11,12,13,14,15,16]. First reason, EUS FNA is not realizable in 6–9% of cases owing to vessel interpositions, duodenal stenosis, and tumoral hardness, particularly in chronic pancreatitis. Then, sensitivity of the EUS FNA is limited

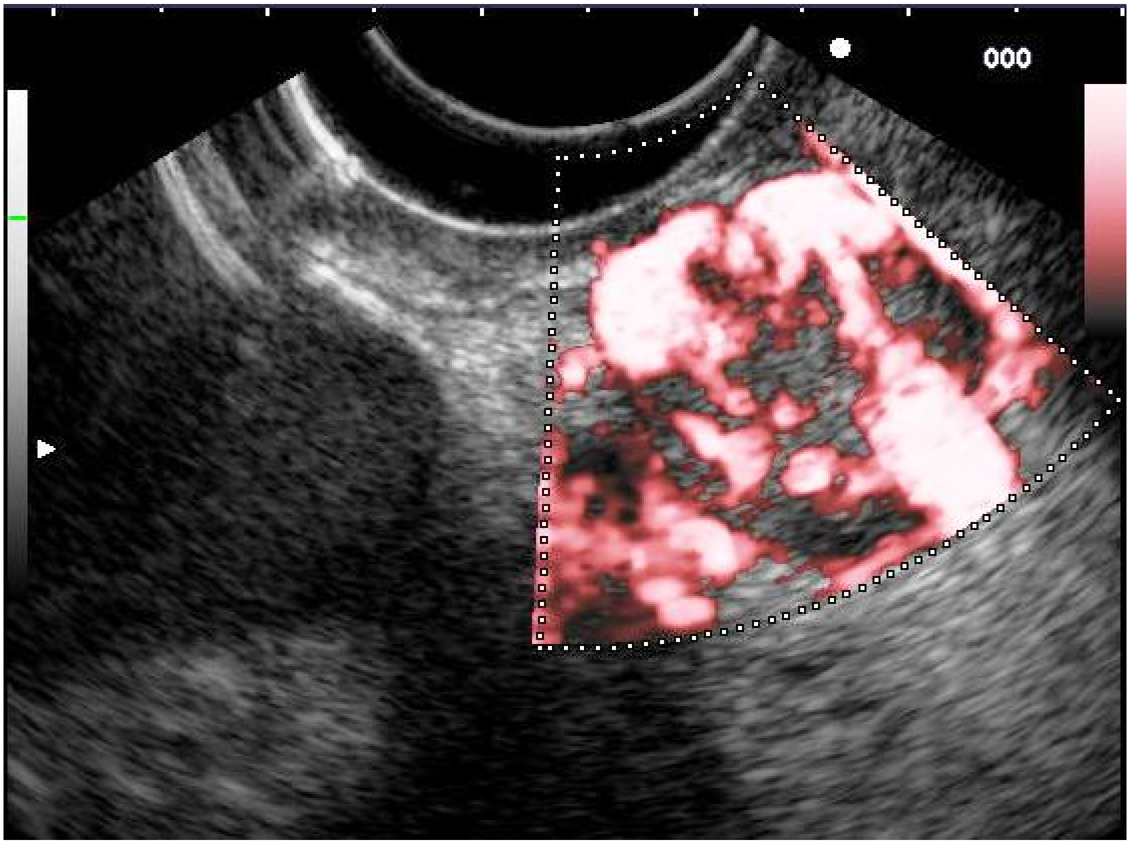

**FIGURE 6B**

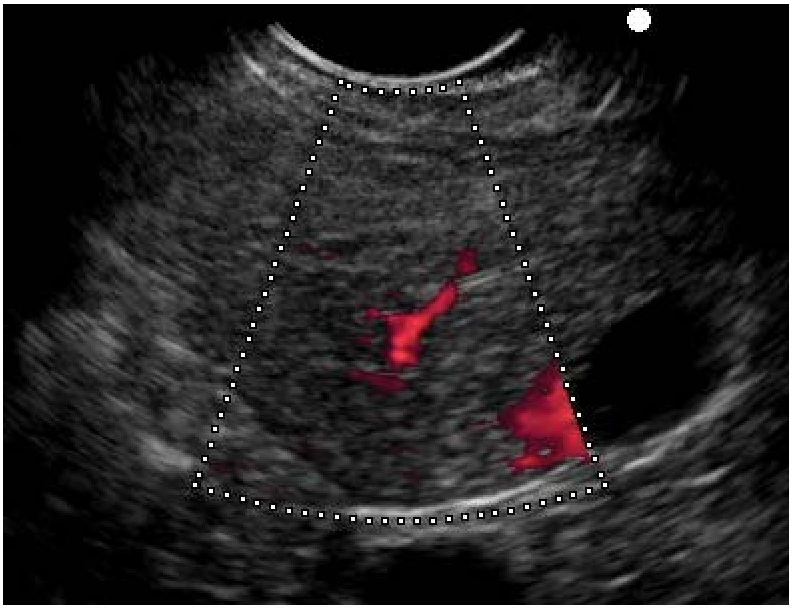

**A**

**FIGURE 7**. (A) Nodule of chronic pancreatitis of the head of pancreas; (B) enhancement of vascularization after sonovue injection.

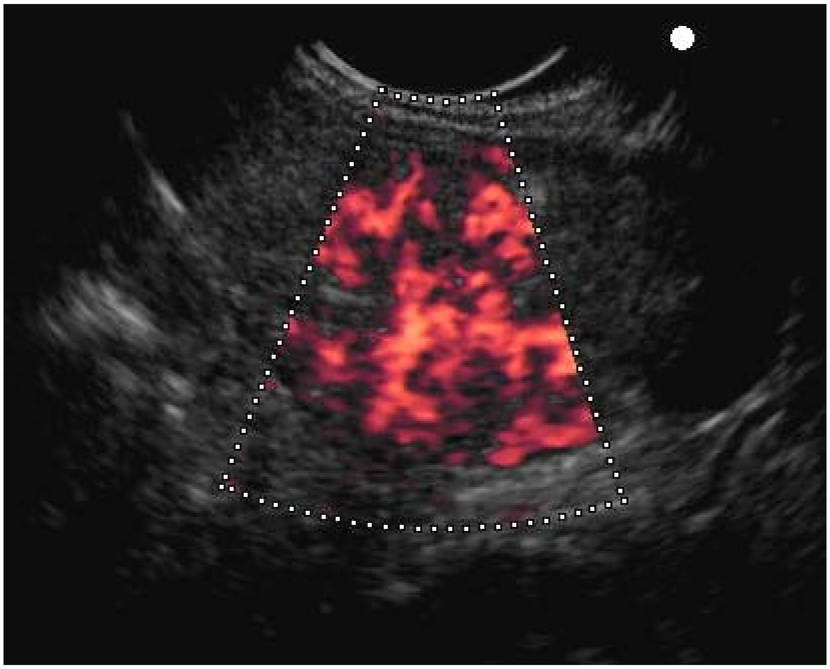

**FIGURE 7B**

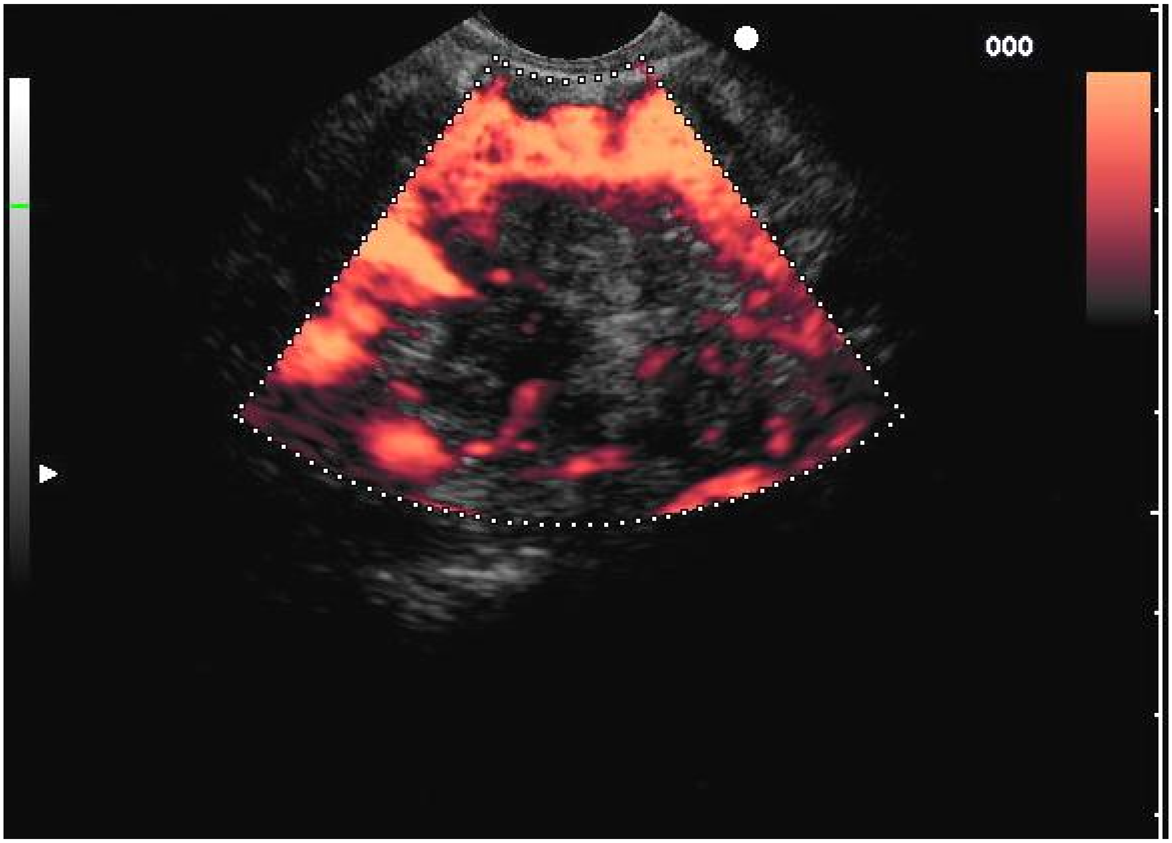

**FIGURE 8**. Pancreatic adenocarcinoma; no enhancement of vascularization after sonovue injection.

by uninterpretable material (bleeding or noncellular samples) ranging from 9–19%. Totally, the lack of sensitivity of EUS FNA ranges from 8–25% of cases[9]. In our work[3], sensitivity and diagnostic accuracy of this technique were comparable to cytopathology results guided by EUS (sensitivity 90.9%, specificity 88.8%, PPV 88.2%, and NPV 91.4%). From a more general point of view, 97% of hypoechoic lesions were malignant tumors (30 adenocarcinoma, 1 endocrine tumor, 1 pancreatic lymphoma, 1 pancreatic metastasis from colonic cancer). Therefore, CE-EUS could appear like a reliable and complementary tool for EUS FNA in detection and classification of pancreatic lesions when EUS FNA is impossible or biopsy uninterpretable. CE-EUS could improve accuracy and allow us to propose an appropriate treatment (surgery, follow-up, chemotherapy, etc.).

CE-EUS could allow us to differentiate malign tumor from pseudotumoral nodule. Chronic pancreatitis is also a limiting factor for diagnosis of pancreatic masses. Several works have attempted to establish EUS imaging criteria (without tissue sampling) for the discrimination of benign inflammatory pseudotumors and tumors. Despite the high resolution of EUS, it does not provide reliable differentiation of benign and malignant lesions of the pancreas[17]. Fritscher-Ravens et al.[19] found that sensitivity of EUS FNA in patients with a focal pancreatic lesion without chronic pancreatitis was 89%, while it was only 54% in patients with chronic pancreatitis. Nevertheless diagnosis of EUS FNA influenced clinical management in nearly half of patients[18]. CE-EUS could also play an important part in the case of lesions occurring within chronic pancreatitis. Indeed, in our study[3], every adenocarcinoma with chronic pancreatitis (3/3; 100%) was hypoechoic after contrast enhancement. Conversely, every pseudotumoral node (benign masses) except one (10/11; 91%) in chronic pancreatitis was hyperechoic. These results are different and better than CE-EUS. For Takeda et al.[20], 100% pseudotumoral pancreatitis had an isoenhanced pattern, and for Takeda et al.[20], it was difficult to differentiate adenocarcinomas from inflammatory pancreatic mass (50% were not well classified).

CE-EUS could be useful in the case of negative results after EUS FNA. In early studies, NPV of EUS FNA was around 75%[13,14], but most recent studies found NPV between 26 and 44%[9,10,11,12, 13,14,15]. In the work from Oshikawa et al.[21], the rate of patients with negative results of the first biopsy, but with malignant tumor diagnosed a second time with a new puncture or with surgery, was 47%. To conclude, negative predictive value of pancreatic EUS-FNA is 30-33%. Theoretically, a new puncture is mandatory to be sure that it is normal tissue. We can also imagine that CE-EUS could avoid this second procedure. With regard to false-negative results of Sonovue®, we found three adenocarcinomas that presented hyperechoic aspect (enhancement contrast pattern). Two were poorly differentiated adenocarcinoma and the third was associated with IMPT. This suggests that poorly differentiated adenocarcinoma could have different vascularity of well-differentiated adenocarcinoma. These results were similar to studies with CE-EUS[22,23]. Differences in histology, such as histological differentiation grade, amount of fibrosis, and obliteration of blood vessels in the tumor, may be associated with differences in enhancement behavior.

Concerning CE-EUS and endocrine tumors, there is only one case report using Levovist that seemed to be a useful diagnostic method for precise localization of small insulinoma[24]. In our study[3], 87.5% (7/8) of endocrine tumors had a strong contrast-enhancement pattern, indicating hypervascular lesions. These results were similar to CE-EUS[19,22,23,25] .These vascular images differed from those of almost all pancreatic ductal carcinomas. Thus, differentiation of enhancement pattern on CE-EUS between pancreatic adenocarcinomas and endocrine tumors is useful in the diagnosis of these lesions. In addition, "standard" EUS is already known to have a great value for localizing endocrine pancreatic tumors because of its excellent capacity to visualize small lesions and tumor vascularization at the same time[26,27]. Therefore, we are authorized to think CE-EUS could increase sensitivity of diagnosis of pancreatic tumors.

Regarding IPMT, in our study[3], the only benign tumor was hyperechoic, whereas in malignant IPMT, one was hypoechoic and another was hyperechoic. In CE-EUS studies, malignancy could be associated with contrast enhancement. For Sofuni et al.[22], all (four patients) with IPMT showed hypervascularity of the nodules inside the tumors. For Nagase et al.[23], two of the five IPMT had solid components within the tumors and they were positive for enhancement effects. All five patients with

IPMT underwent surgical resection and pathologic examination revealed malignancy in the two lesions with solid components and positive enhancement. For Itoh et al.[28], when the patients with carcinoma were compared with those with adenoma, the postenhancement intensity was significantly higher in the carcinoma group. CE-EUS could be useful for the differential diagnosis of benign and malignant IPMT. The small number of patients with IMPT in each study did not allow conclusions.

Metastatic lesions of the pancreas are rare, between 5–10%[29], but an important cause of focal pancreatic lesions (Figs. 9A and B). There is only one description of one case of kidney metastasis analyzed in CE-EUS[30]. Our work[3] is the first in the literature that describes the enhancement pattern of pancreatic metastasis in CE-EUS. All metastasis except one (4/5; 80%) showed an echo enhancement pattern, probably proving their hypervascularization. The only pancreatic metastasis nonenhanced was from colonic cancer. CE-EUS could provide a contribution to the differential diagnosis between a primary pancreatic carcinoma and a pancreatic metastasis, and therefore can have a decisive influence on the selection of appropriate therapeutic strategies (chemotherapy rather than surgery, for example). However, histology remains the standard in the differential diagnosis of pancreatic tumors.

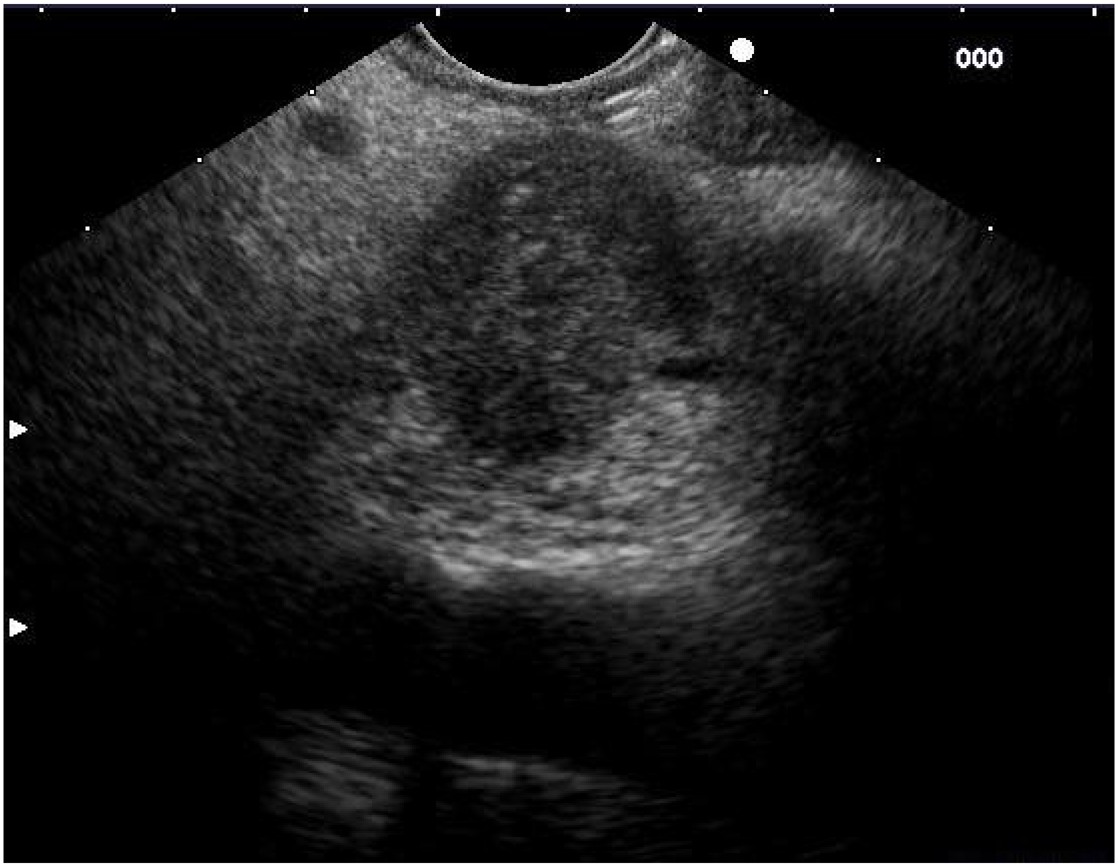

A

**FIGURE 9**. (A) Renal metastasis of the head of pancreas; (B) enhancement of vascularization after sonovue injection.

## EUS SONOELASTOGRAPHY

The introduction of EUS represented a major advance in the diagnosis and staging of gastrointestinal malignancies. In addition to providing imaging of tumors and enhancing TMN (Malignancy (large node on Ct)) staging, EUS also provides guidance for FNA and biopsies of undiagnosed masses and lymph nodes (LN) suspicious for malignant

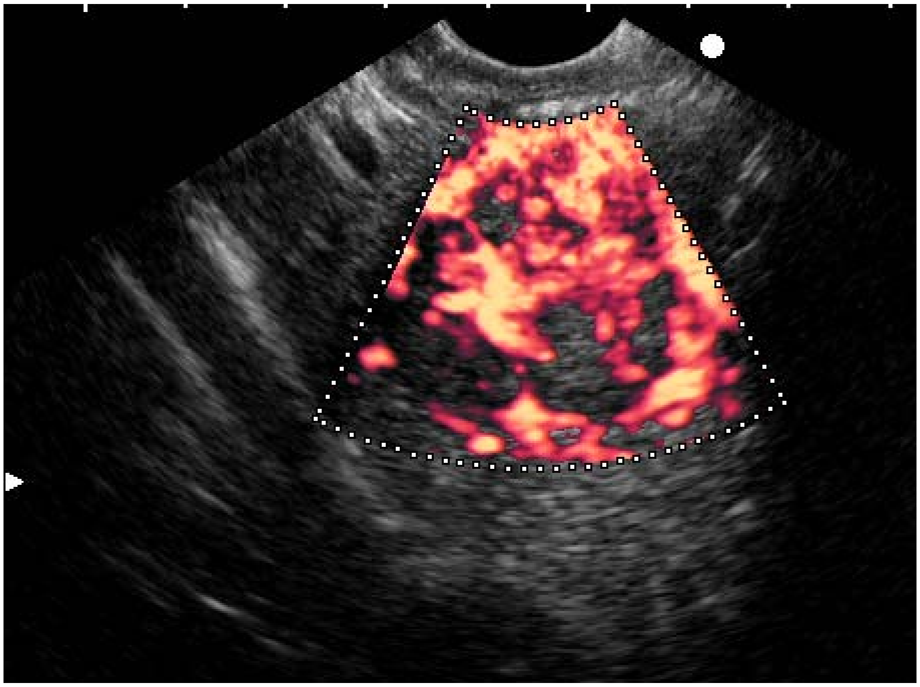

**FIGURE 9B**

invasion, providing further diagnostic and staging information. However, FNA is technically demanding and multiple punctures of LN or masses are sometimes required to obtain sufficient tissue for histologic assessment. In addition, when several LN appear suspicious, the choice of which to puncture is not always clear. Current sonographic criteria for malignant LN (round, hypoechogenicity, diameter >1 cm, and distinct margins) are helpful in targeting lesions, but problems exist with specificity and overlap of these features with benign LN[31,32]. For further consideration is the fact that pancreatic masses have a wide differential diagnosis that includes benign and malignant etiologies, and FNA of the pancreas is associated with a small, but not insignificant, risk of pancreatitis[33]. Hence, the ability to evaluate masses and LN more accurately prior to their puncture in an effort to aid in targeting lesions for FNA and possibly reduce complications would be welcomed by echoendoscopists. At least two strategies have been developed with these goals in mind: contrast-enhanced endosonography and sonoelastography.

### Theory and Technical Aspects of Sonoelastography

Sonoelastography is based on the knowledge that some diseases, such as cancer, lead to a change of tissue hardness (i.e., the so-called elasticity modulus) and is an outgrowth of the well-known breast ultrasound fremitus technique[34,35,36], during which the patient is asked to hum while color or power Doppler is used to examine the breast. Softer portions of the breast vibrate more in response to the humming, while cancers and other firm masses vibrate less and thus are seen as areas of decreased color, even if they are isoechoic on the ordinary B scan. Elastography examines the elastic properties of tissues by applying a slight compression to the tissue and comparing an image obtained before and after this compression. The before and after data are then compared, using a cross-correlation technique to determine the amount of displacement each small portion of tissue undergoes in response to the compression applied by the US transducer[37,38,39]. The elasticity modulus, i.e., the tissue elasticity distribution, can be calculated from the strain and the stress of the examined structures. While the strain field can be estimated from the radio frequency signals returned from tissue structures before and after compression, it is impossible to measure the stress field directly within the tissue. Another problem is that the compression of harder tissue structures is often followed by a lateral displacement of these structures[40]. It is nearly impossible to represent the volume of this sideslip with conventional 2-D methods, but its calculation is indispensable for an accurate determination of the tissue elasticity of the examined structures. To overcome these problems, the Extended Combined Autocorrelation Method has been developed, which allows the reconstruction of the tissue elasticity of the examined structures on the basis of the 3-D finite element model. The new technique enables highly accurate estimation of the tissue elasticity distribution and adequate compensation of sideslips. The elasticity imaging can be performed in real time with the *SonoElastography module* that can be integrated into the platform of the HITACHI EUB-8500 system (Hitachi Medical Systems Europe, Zug, Switzerland).

## Procedure Technique and Criteria

As with traditional color Doppler imaging, EUS tissue elasticity imaging is performed with conventional EUS probes and does not require additional instruments (e.g., for measuring pressure or producing vibrations). The vibrations and compressions are provided physiologically by vascular pulsation and respiratory motion. The calculation of tissue elasticity distribution is performed in real time and the examination results are represented in color superimposed over the conventional B-mode image.

To date, the majority of clinical research involving sonoelastography has been focused on the evaluation of breast masses. Three different patterns have been identified in elastograms of breast cancers: a well-defined, very hard (dark) mass or nodule; a moderately hard mass or nodule containing much harder (darker) foci within it; and a very dark or hard central core surrounded by a somewhat softer or less dark peripheral component[36]. Although with conventional US or EUS fibrosis generally appears as hyperechogenic regions with posterior acoustic shadowing (an appearance also seen in cancers), in elastography, it generally appears as a uniform, moderately hard region with no distinct foci of increased hardness. Preliminary work in breast tissue elastography has shown that it can correctly classify most benign and malignant masses[36].

### *EUS Sonoelastography*

We have published recently our preliminary experience on 49 patients[41]. Between March 2004 and April 2005, 49 patients underwent EUS examination with sonoelastography. Indications for elastography examination included evaluation of a pancreatic mass (n = 24) and assessment of suspicious LN (n = 25). The real-time elasticity imaging described in this study was performed with the SonoElastography module that was integrated into the platform of the HITACHI EUB-8500 system (Hitachi Medical Systems Europe, Zug, Switzerland). Like color Doppler examinations, tissue elasticity imaging was performed with EUS-scope EG 38-UT (Pentax Europe Gmbh, Hamburg, Germany) and does not require additional instruments. The examination results are represented in color over the conventional B-mode image with malignant tissue appearing in blue, fibrosis in green, normal tissue in yellow, and fat in red.

A EUS FNA was performed in all cases using a 22-gauge needle (Wilson-Cook Medical, Winston-Salem, NC). As per usual in our center, all specimens are examined using the monolayer technique and although no cytopathologist is present when biopsies are performed, the endoscopist assesses the sample to ensure adequate tissue core, with repeat punctures if necessary.

Masses or LN that appeared mostly blue (harder) were considered malignant, with other results considered benign. Final diagnosis was based on histology from FNA and surgical specimens when available.

Results are presented as mean with standard deviation or median with range, depending on data distribution.

## Pancreatic Masses

Twenty-four patients (median age 60 years [range 39–88]) underwent EUS examination with sonoelastography for evaluation of a pancreatic mass (mean diameter 24.7 mm ± 11.1). Masses were located in the pancreatic head (n = 12), body (n = 6), and tail (n = 6). Final histology was based on FNA results in 21 cases and surgical pathology in 3 cases. Final diagnosis of malignant masses included adenocarcinoma of the pancreas (n = 14), metastatic renal cancer (n = 2), sarcoma (n = 1), and ovarian cancer (n = 1). Benign masses consisted of chronic pancreatitis–related nodule (n = 4), neuroendocrine tumor (n = 1), and an IPMT (n = 1).

Sonoelastography images of pancreatic masses were interpreted as benign in 4 cases and malignant in 20. Two masses were misclassified as malignant by elastography; the first was a neuroendocrine tumor, and in the second case, the patient underwent surgical resection and final pathology revealed the mass to be a benign fibromyoblastic tumor of the pancreas. Sensitivity and specificity of sonoelastography in the diagnosis of malignant lesions was 100 and 67%, respectively.

A subsequent review of our experience with elastography and pancreatic masses was performed and a more refined classification of pancreatic mass sonoelastography images has been developed in which EUS elastography images are differentiated into five scores (Fig. 10). Score 1 is for a homogenous, low elastograph area (soft, green) and corresponds to the normal pancreas tissue (Fig. 11). For images classified as Score 2, there is heterogenicity of the elastograph, but in the soft tissue range (green, yellow, and red), and corresponds to fibrosis (Fig. 12). Score 3 is for an elastograph image that is largely blue (hard) with minimal heterogenicity and corresponds to a small, early (less than 25 mm) pancreatic adenocarcinoma (Fig. 13). In tumors assigned Score 4, there exists a hypoechoic region in the center of the tumor, with green appearance for this small area surrounded by blue or harder tissue, and corresponds to a hypervascular lesion (Fig. 14), such as a neuroendocrine tumor or small pancreatic metastasis. Finally, Score 5 is assigned to lesions that are largely blue on elastograph, but with heterogenicity of softer tissue colors (green, red), representing necrosis, and is seen in advanced pancreatic adenocarcinoma (Fig. 15).

## Lymph Nodes

Twenty-five patients (median age 57 years [range 16–76]) underwent EUS examination with sonoelastography of 31 LN. The mean diameter of the LN was 19.7 mm ± 8.6 and were found in the cervical area (n = 3), mediastinum (n = 17), celiac arterial trunk region (n = 5), and aortocaval region (n = 6). Final histology was based on FNA and concluded the LN to be benign in 14 cases and malignant in 17. Sonoelastography images of the LN were interpreted as malignant in 22 cases (Figs. 16A and B), benign in 7 (Figs. 17A and B), and indeterminate in 2. While there were no false-negatives, there were five false-positives. The indeterminate cases were due to heterogenicity of the sonoelastograph images and were both benign on final histology. The sensitivity and specificity of sonoelastography in the evaluation of malignant invasion of LN was 100 and 50%, respectively. Six patients had sonoelastography of more than one LN. In two of these cases, one LN was benign and the other malignant, and elastography correctly differentiated between the two.

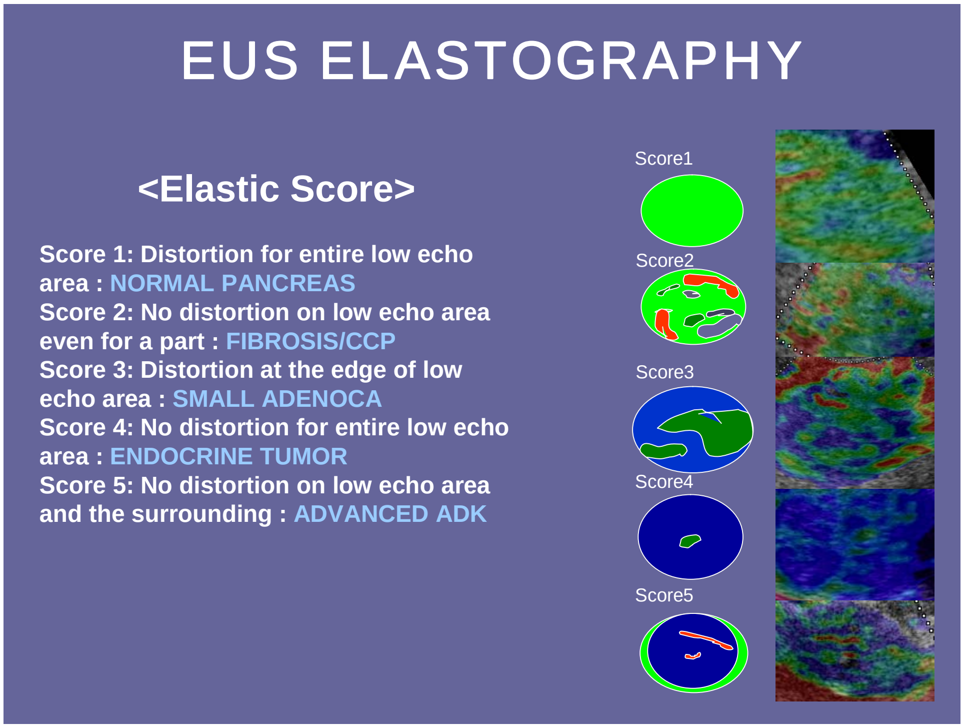

**FIGURE 10**. Elastography score for pancreatic masses.

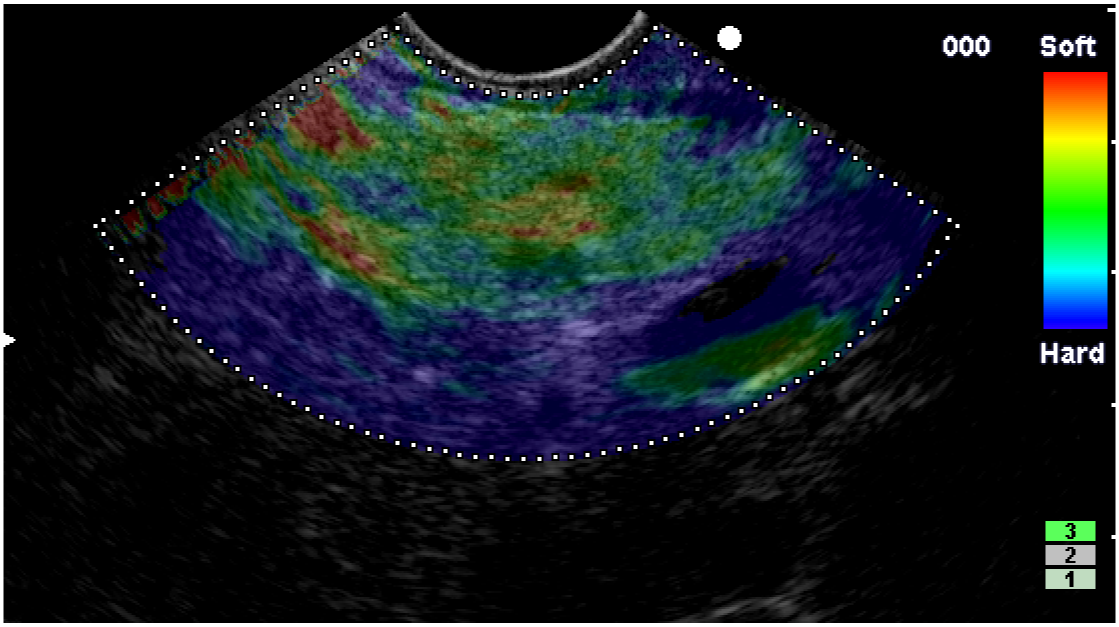

**FIGURE 11**. Score 1: normal pancreatic tissue.

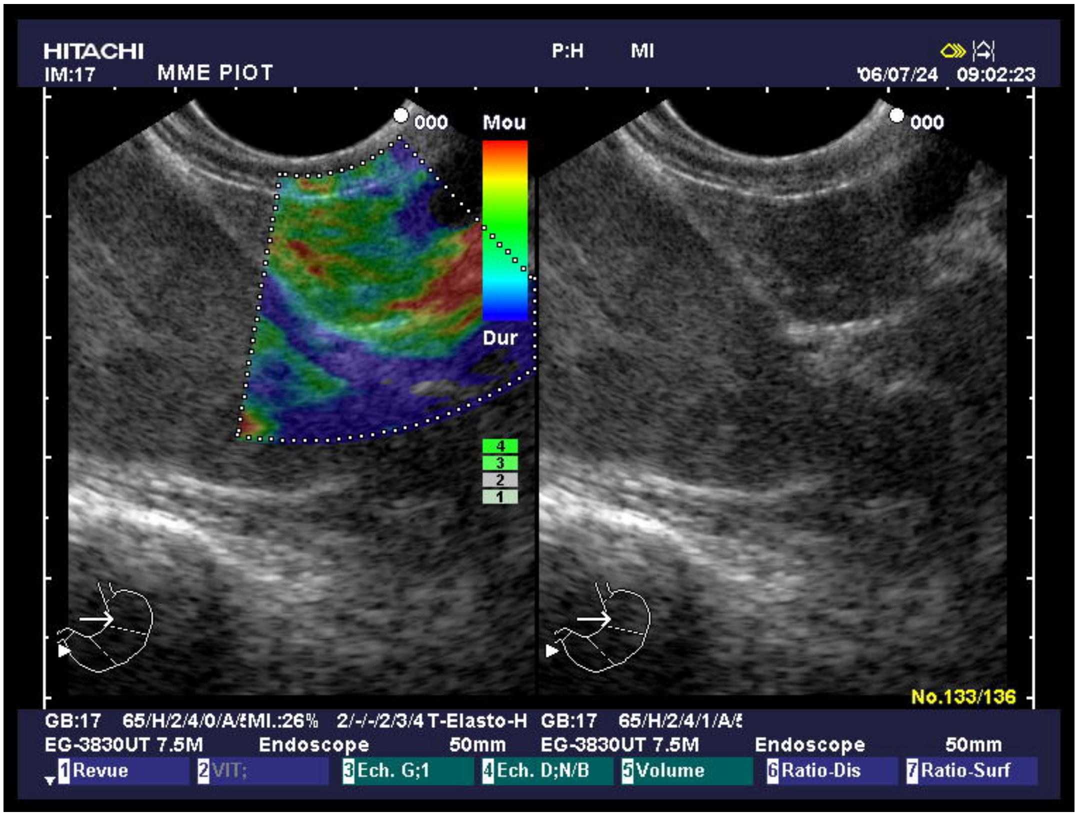

**FIGURE 12.** Score 2: nodule of chronic pancreatitis.

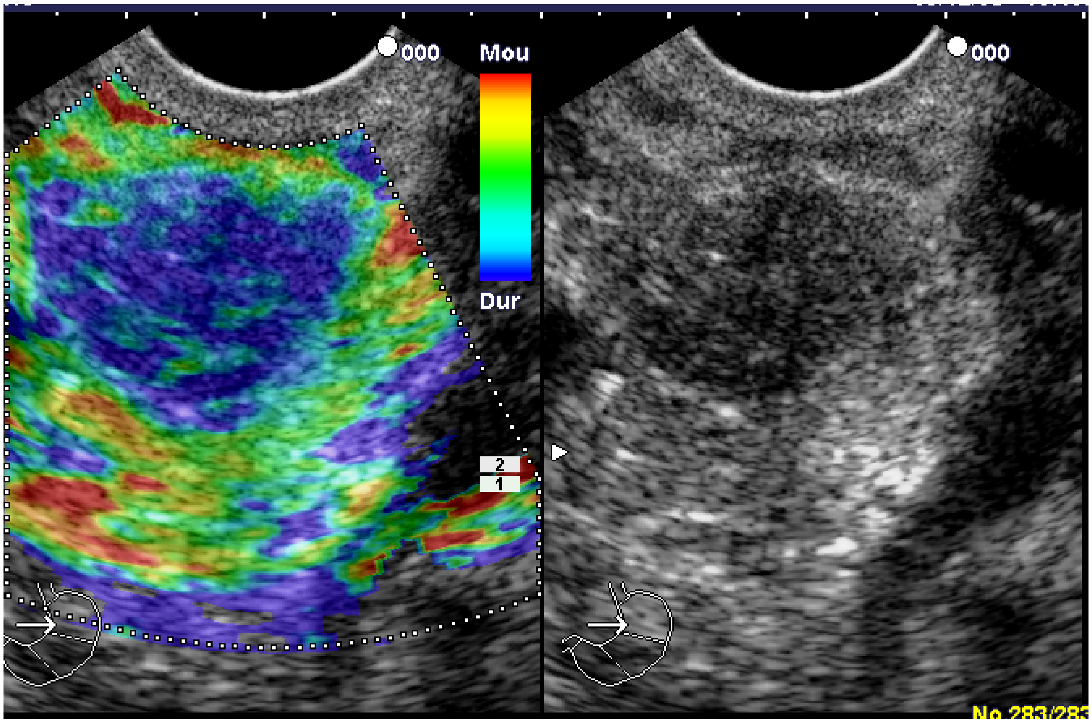

**FIGURE 13.** Score 3: small adenocarcinoma of the pancreas.

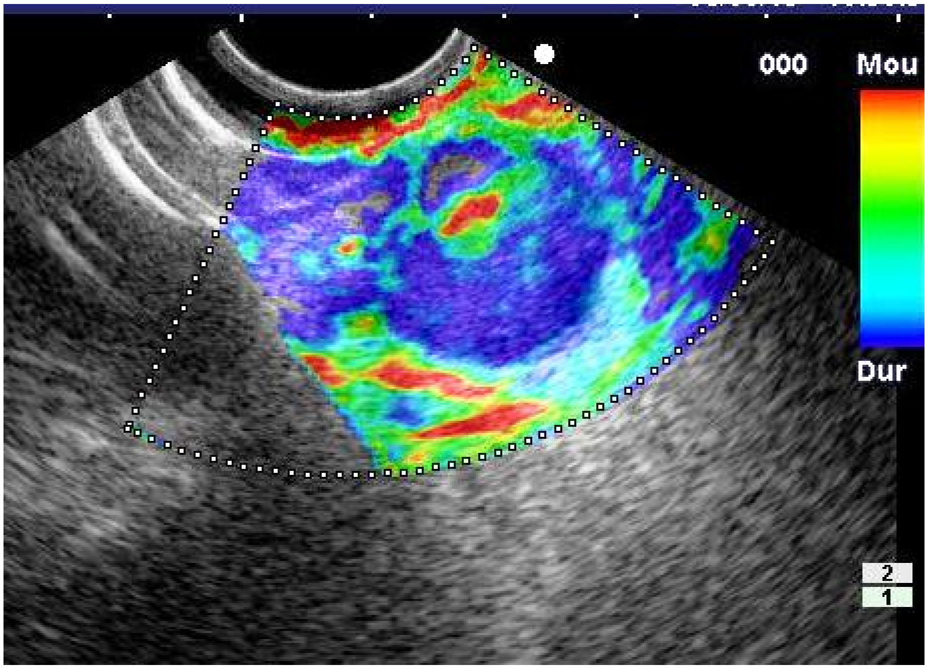

**FIGURE 14.** Score 4: malignant endocrine tumor of the head of pancreas.

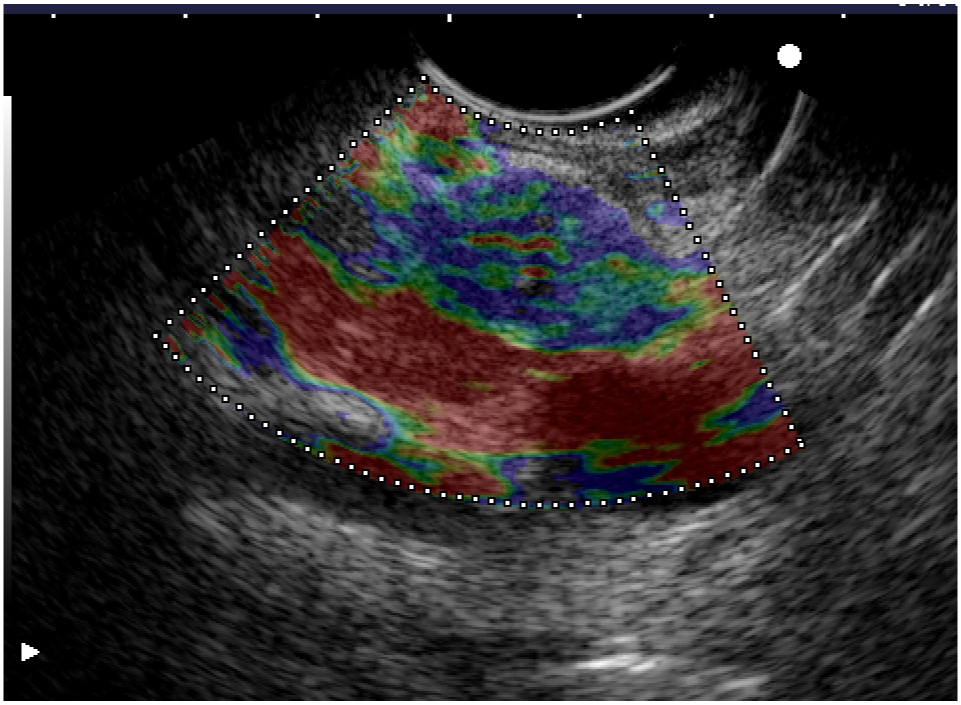

**FIGURE 15**. Score 5: pancreatic adenocarcinoma developed on chronic pancreatitis.

The elasticity of soft tissues depends to a large extent on their molecular building blocks (fat, collagen, etc.), and on the microscopic and macroscopic structural organization of these blocks. In the normal pancreas, for example, the glandular structure may be firmer than the surrounding connective tissue, which in turn is firmer than the subcutaneous fat. Sonoelastography allows the hardness or stiffness of biological tissues to be estimated and imaged using conventional US instruments with modified software. It is known that certain pathologic conditions, such as malignant tumors, often manifest themselves as changes in the mechanical properties of tissue. We believe that the elastic properties of benign lesions are fairly uniform, such as throughout a benign tumor. Cancerous tumors, on the other hand, grow in a very disorganized way. Therefore, within a given malignant tumor, the elastic properties of one area of a tumor may be significantly different from those in another area. The concept relating to the measurement of these tissue changes is an extension of the basic principles associated with traditional medical US imaging. The principle is based on the fact that tissues are deformed slightly when a small displacement is externally applied[38,39,42].

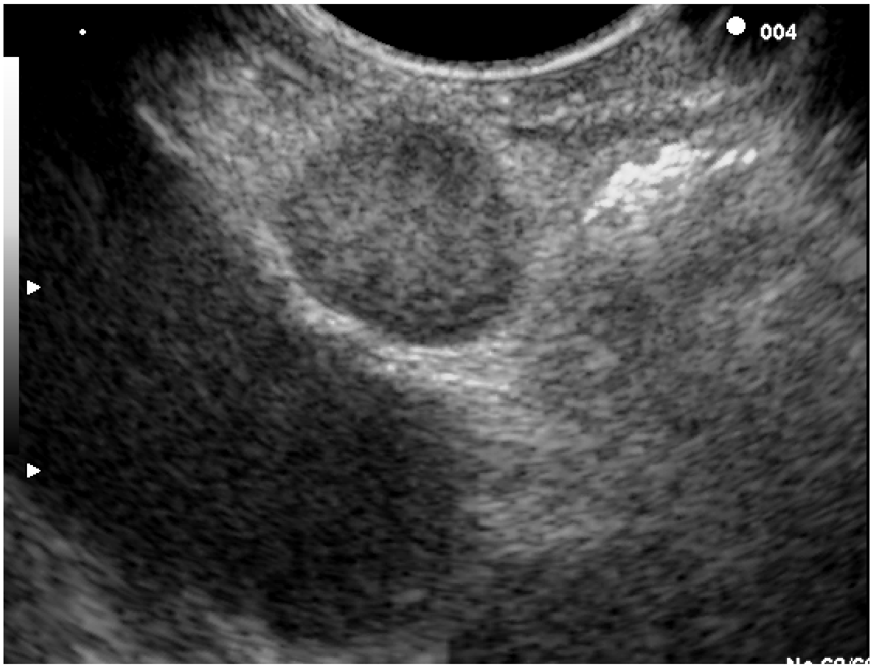

**A**

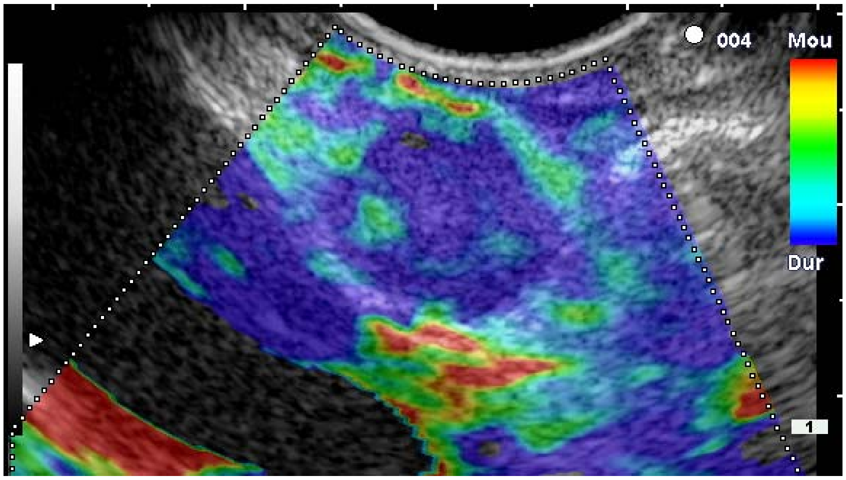

**B**

**FIGURE 16**. (A) Pericaval LN; (B) elastography: malignant LN: Score 4.

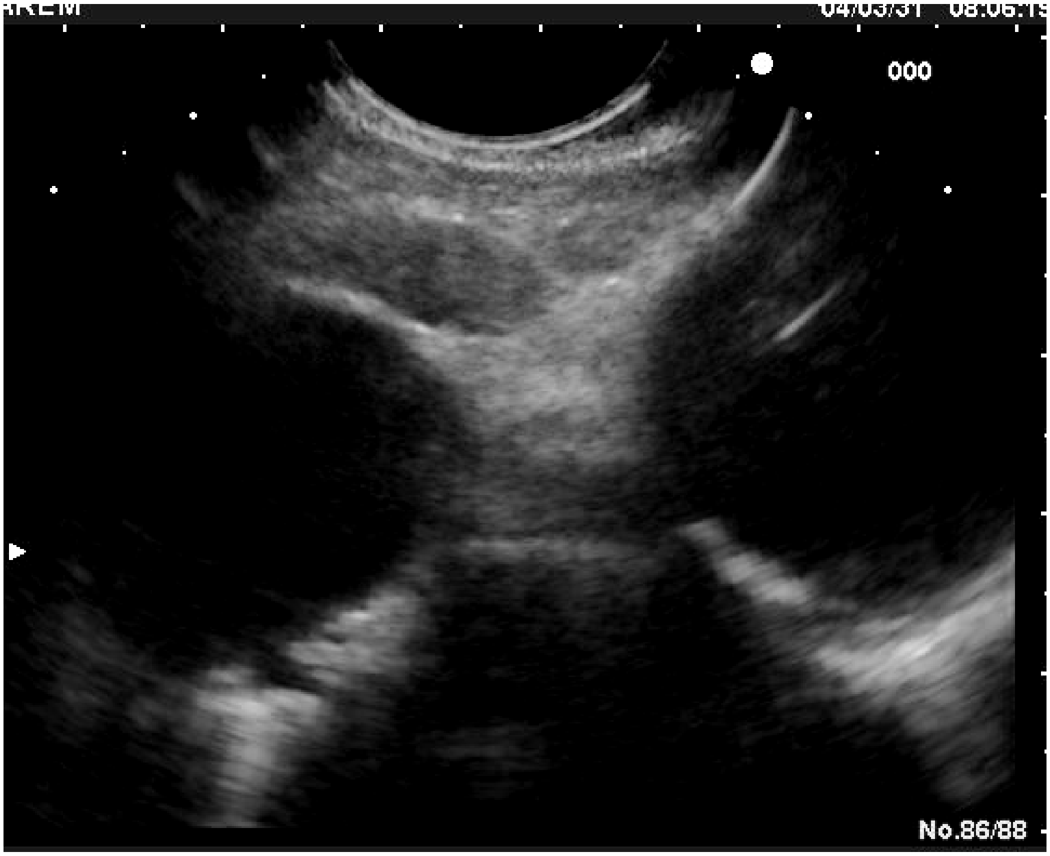

A

**FIGURE 17.** (A) Mediastinal LN; (B) elastography: inflammatory LN: Score 2.

This work represents an extension of our previous results of EUS-guided sonoelastography[43]. The sensitivity in assessment of both pancreatic masses and LN was 100%. Although there were false-positives in both patient groups, and concerns may exist regarding the specificity of sonoelastography in both settings (80% in pancreas group, 50% in LN group), one must recall that the number of benign lesions in this study was relatively small. It is likely that with more experience and refined criteria, the specificity will improve. In fact, we have reviewed our experience with elastography and pancreatic masses, and have developed a new, more-refined classification of sonoelastography images (Fig. 10). Further assessment of this system is ongoing. The results in the six patients with multiple suspicious LN highlight the potential utility of elastography, which is the selection of which LN(s) to puncture, thus potentially reducing puncture-related risk and reducing procedure time. Although EUS-guided FNA has the potential to miss microinvasion of malignancy into LN, and thus represents a somewhat imperfect gold standard, in the absence of surgical specimens, we feel it is representative of daily practice, particularly when combined with an adequate follow-up period.

## *Results of a Prospective Multicenter Study on Pancreatic Masses (Fig. 18)*

Between October 2005 and February 2006, 121 patients (77 male and 44 female), mean age 63 years, underwent EUS for a pancreatic mass. The final diagnosis was obtained by EUS FNA in 82 cases and by

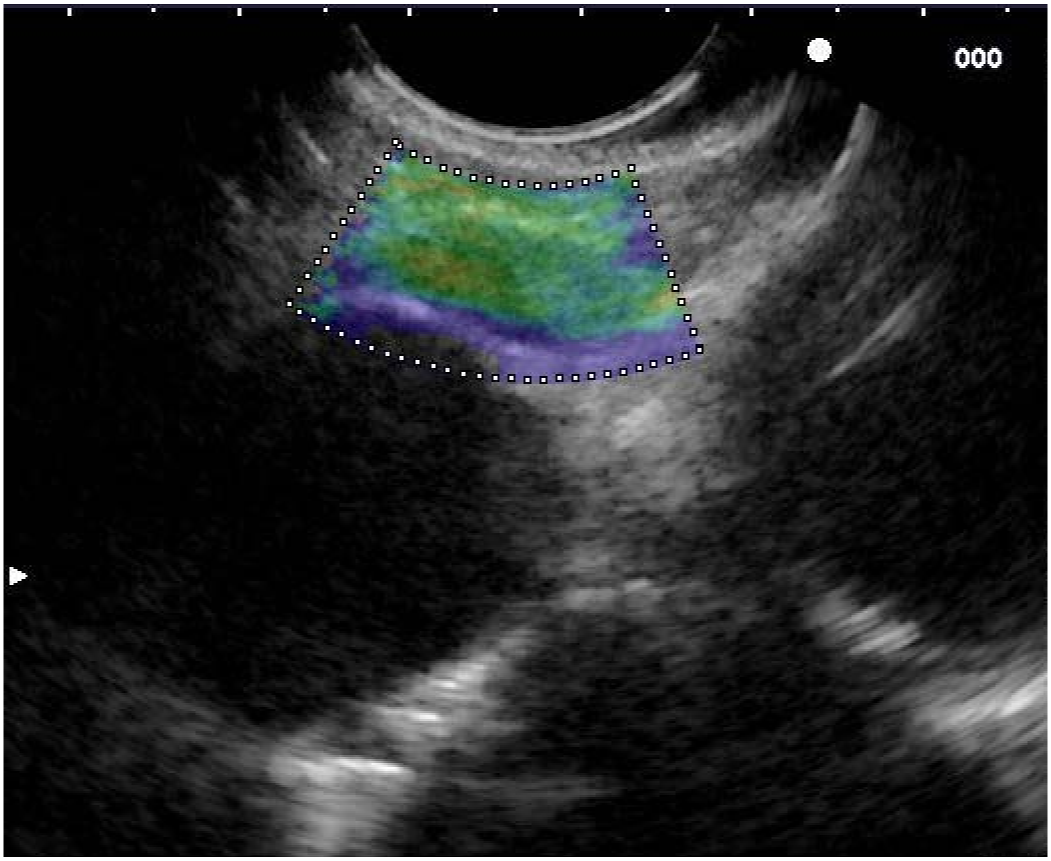

**FIGURE 17B**

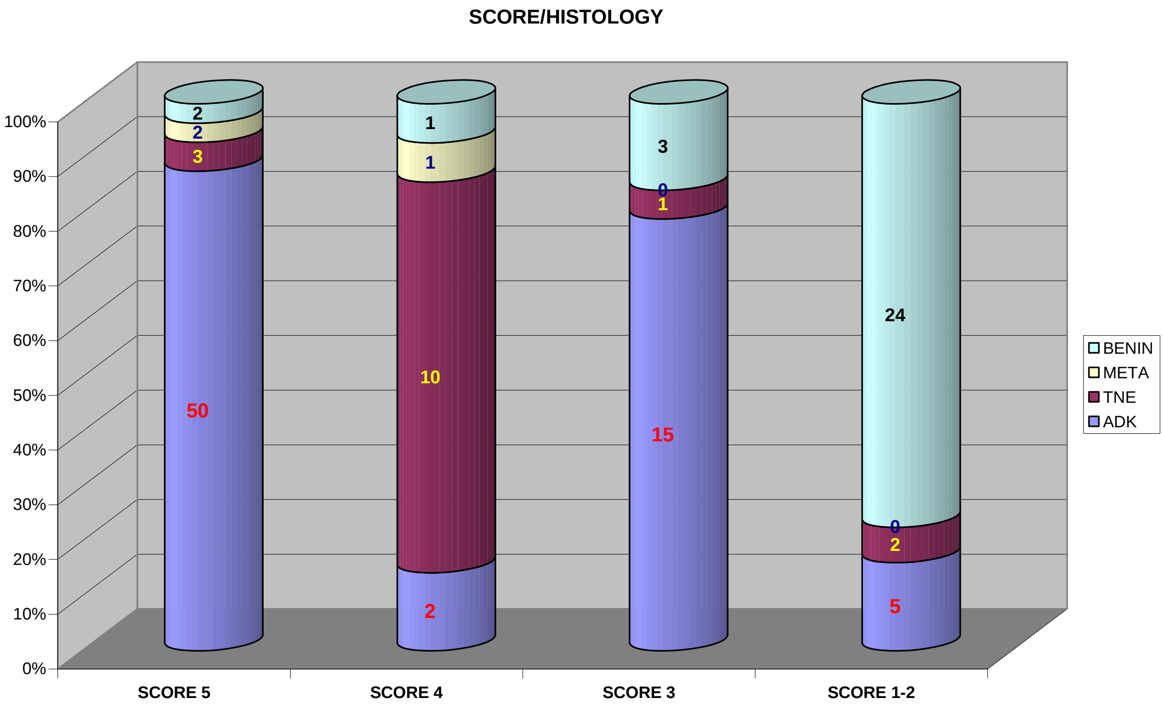

**FIGURE 18**. Multicentric study: correlation between elastography score and histology for pancreatic masses.

surgery in 39 cases. Final histology was pancreatic adenocarcinomas (72 cases), endocrine tumors (16 cases), benign nodule of chronic pancreatitis (30 cases), and pancreatic metastasis (3 cases). Elastography showed malignant aspects (intense blue coloration) for all pancreatic adenocarcinomas, endocrine tumor, pancreatic metastasis, and pancreatic sarcoma. All nodules of chronic pancreatitis presented benign aspects (mixed green and low intensity of blue). The LN sonoelastography classification was done in 5 scores (1–5). If we considered the scores 1 and 2 as benign, and scores 3–5 as malignant, the sensibility, specificity, PPV and NPV of EUS sonoelastography to differentiate benign from malignant pancreatic masses were, respectively, 80.6, 92.3, 93.3, and 78.1% with a global accuracy of this new technology of 89.2%. The NPV for malignancy of scores 1–2 was 77.4% and the PPV for malignancy of scores 3–5 was 92.8%. An interobserver study on 30 patients showed a good concordance (kappa score = 0.7) for the diagnosis of malignant pancreatic masses using sonoelastography.

### Results of a Prospective Multicenter Study on Lymph Nodes Staging

Between October 2005 and February 2006, 101 patients (56 male and 45 female), mean age 61.1 years, underwent EUS FNA of LN for staging of lung cancer (26 cases), esophageal carcinoma (25 cases), gastric cancer (13 cases), pancreatic cancer (12 cases), for a suspicion of LN relapse of a kidney cancer (2 cases), and of a breast cancer (8 cases). A EUS FNA was also performed in 15 cases for isolated LN. LN were located in the mediastinum (51 cases), the cervical area (4 cases), the celiac or mesenteric area (44 cases), and perirectal space (2 cases). The mean size of the lesion was 20.1 mm (range: 7–50 mm).

Final histology was malignant LN (55 cases including 35 metastasis by an adenocarcinoma, 13 by a squamous cell carcinoma, 3 by an endocrine tumor, 1 melanoma, and 5 lymphomas) and inflammatory LN (44 cases including 3 cases of sarcoidosis). The LN sonoelastography classification was done in 5 scores (1–5). If we consider scores 1–3 as benign and scores 4 and 5 as malignant, the sensibility, specificity, PPV and NPV of EUS sonoelastography to differentiate benign from malignant LN were, respectively, 100, 83.3, 100, and 75%. But, if we consider scores 1 and 2 as benign and scores 3–5 as malignant, the sensibility, specificity, PPV and NPV of EUS sonoelastography to differentiate benign from malignant LN were, respectively, 88.10, 88.13, 91.22, and 84.10% with an accuracy of this new technique between 88 and 89.10%.

## CONCLUSIONS

EUS elastography is a new application in the field of endosonography and seems to be able to differentiate fibrous and benign tissue from malignant lesions. While our results are very encouraging, further research will be needed to further define the place of this new technique and should be aimed at further defining criteria for accurate elastography as well as subsequently assessing the technique using multiple operators in a blinded setting. EUS-guided sonoelastography has the potential for further guiding the diagnosis and therapy of gastrointestinal-related tumors.
